# TGF beta receptor II interacting protein-1, an intracellular protein has an extracellular role as a modulator of matrix mineralization

**DOI:** 10.1038/srep37885

**Published:** 2016-11-24

**Authors:** Amsaveni Ramachandran, Sriram Ravindran, Chun-Chieh Huang, Anne George

**Affiliations:** 1 Brodie Tooth Development Genetics & Regenerative Medicine Research Laboratory, Department of Oral Biology, University of Illinois at Chicago, Chicago, Il 60612, USA

## Abstract

Transforming growth factor beta receptor II interacting protein 1 (TRIP-1), a predominantly intracellular protein is localized in the ECM of bone. TRIP-1 lacks a signal peptide, therefore, in this study, we provide evidence that intracellular TRIP-1 can be packaged and exported to the ECM via exosomes. Overexpression of TRIP-1 in MC3T3-E1 cells resulted in increased matrix mineralization during differentiation and knockdown resulted in reduced effects. *In vivo* function of TRIP-1 was studied by an implantation assay performed using TRIP-1 overexpressing and knockdown cells cultured in a 3-dimmensional scaffold. After 4 weeks, the subcutaneous tissues from TRIP-1 overexpressing cells showed higher calcium and phosphate deposits, arranged collagen fibrils and increased expression of Runx2 and alkaline phosphatase. Nucleation studies on demineralized and deproteinized dentin wafer is a powerful tool to determine the functional role of noncollagenous proteins in matrix mineralization. Using this system, we provide evidence that TRIP-1 binds to Type-I collagen and can promote mineralization. Surface plasmon resonance analysis demonstrated that TRIP-1 binds to collagen with *K*_*D*_ = 48 μM. SEM and TEM analysis showed that TRIP-1 promoted the nucleation and growth of calcium phosphate mineral aggregates. Taken together, we provide mechanistic insights of this intracellular protein in matrix mineralization.

TRIP-1 is differentially regulated during osteoblast maturation and is more abundantly expressed during the early stages of this process^1^,^2^. During bone remodeling, TRIP-1 expression is regulated by hormones and growth factors that are known to affect bone formation. Recent studies on TRIP-1 show its role in osteoblast proliferation and differentiation^1^. This is supported by the fact that TRIP-1 acts as a positive regulator of TGFβ signaling and has a cell specific role through its interaction with tartrate-resistant acid phosphatase (TRAP)^3^.

Several studies have shown elevated expression levels of TRIP-1 expression in various human cancers, including breast tumor, head and neck squamous cell carcinomas, and HCC tissues^4^,^5^,^6^. Recent studies on the functional role of TRIP-1 in cancer have suggested that TRIP-1 functions as a proto oncogene^7^. Recent studies by Wang *et al.*^8^ have shown that clusterin interacts with TRIP-1 to activate Akt pathway, promoting expression of MMP13 which leads to metastasis of HCC cells.

TRIP-1, also known as eIF3i is a subunit of eIF3 (eukaryotic initiation factor-3) which plays a role in translation regulation, cell growth and cancer^9^. Deletion studies showed that the rate of the protein synthesis does not change by knockdown of the evolutionarily conserved i-subunit of eIF3 (eIF3i)^10^. Therefore, it is suggested that eIF3i is not essential for eIF3 activity but might be involved in translational control of specific mRNAs or in particular cellular conditions^11^.

The ECM of bone and dentin contain a complex network of macromolecules with distinctive physical, biochemical and biomechanical properties^12^. Although the organic matrix is predominantly comprised of collagen fibrils, it also contains a diverse array of noncollagenous proteins^13^. Disruption of ECM dynamics may contribute to several mineralization-related disorders. In biominerals, the small amount of organic matrix, not only reinforces the mechanical properties of the biocomposite, but also contributes to the determination of the size, crystal morphology and specific crystallographic orientation^14^. Thus, the inorganic-organic hybrid composites attain the superior mechanical properties by self-assembled bottom-up processes. Therefore, understanding the ECM composition and their influence on calcified tissue formation is necessary to understand the normal development of bone and dentin^15^,^16^.

We have reported earlier that TRIP-1 is localized in the mineralized matrices of bone and dentin^17^. Localization studies show that TRIP-1 is expressed in osteoblast and odontoblasts^1^,^7^,^17^. However, TRIP-1 mRNA and protein expression was found to increase during early phase of osteogenic differentiation and decreased during the later phase, suggesting a functional role during early maturation of the osteoblasts^17^. In the cartilage, TRIP-1 was expressed in the proliferating chondrocytes and with development of the growth plate, the expression of TRIP-1 was confined only to the primary ossification center^17^. Thus, the presence of secreted TRIP-1 in the ECM of bone, dentin and cartilage clearly demonstrates the diversity of proteins that exists in the calcified matrix.

In this current study, we have demonstrated that the calvarial preosteoblasts express TRIP-1 and high levels were observed in the secretome of primary calvarial osteoblasts during mineralization. Further, we have explored the mode of transport of TRIP-1 to the extracellular matrix and also investigated the role of TRIP-1 in the ECM.

## Results

### TRIP-1 expression during the differentiation of calvarial osteoblasts

**Figure 1:**
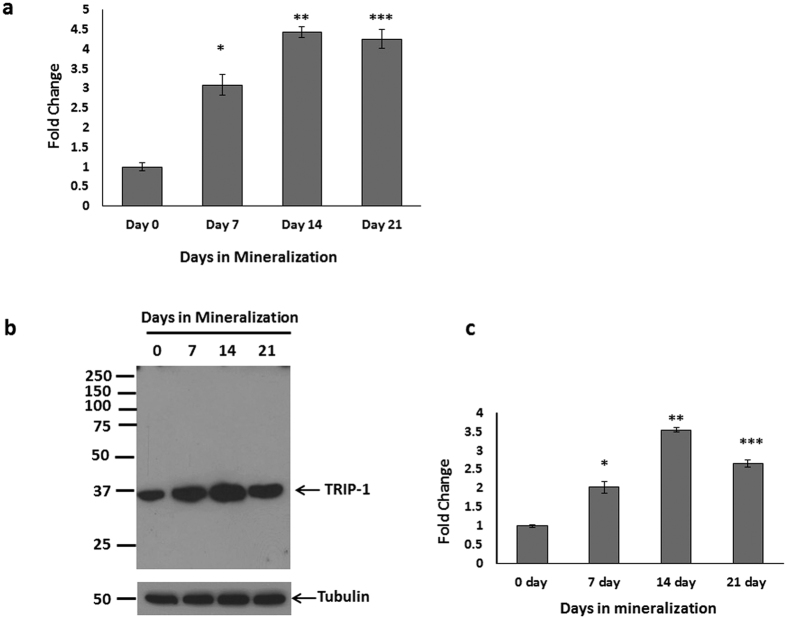
Expression of TRIP-1 during osteoblast differentiation. (**1a**) Mouse primary calvarial osteoblasts were cultured in differentiation media and total RNA was isolated at 0, 7, 14 & 21 days and real-time PCR analysis was performed. The expression of TRIP-1 increased significantly during differentiation up to 21 days. (**1b**) Western Blot analysis of total protein from primary mouse calvarial osteoblasts showing expression of TRIP-1 during differentiation. Tubulin was used as a loading control. (**1c**) Quantitative analysis of TRIP-1 protein expression. Data are represented as mean fold change obtained from triplicate experiments with standard deviation as error. Student’s *t*-test used to obtain statistical significance with respect to control. *, **, *** represents significance of p < 0.05.

Real-Time PCR analysis was performed on mRNA isolated from primary calvarial osteoblast cells cultured under differentiation conditions for 7, 14 & 21 days while 0 day served as control. RT PCR quantitation showed increasing expression of TRIP-1 up to 21 days when compared to control (Fig. 1a). Western blot analysis of the total protein (Fig. 1b) showed a similar expression pattern. Quantitative analysis (Fig. 1c) shows the increase in TRIP-1 protein expression is statistically significant (*, **, ***p < 0.05). This suggests that TRIP-1 expression correlated well with osteoblast differentiation. To determine if TRIP-1 is expressed abundantly in other cell types, western blot analysis was done on total proteins isolated from C2C12 muscle cell line, MDA-231 breast cancer cells and HMSCs. C2C12 and HMSCs cells showed increased expression than MDA-231 cells (Supplementary Figure S1).

### TRIP-1 is a secreted protein and is present in the secretome of primary osteoblasts and in the extracellular matrix

**Figure 2:**
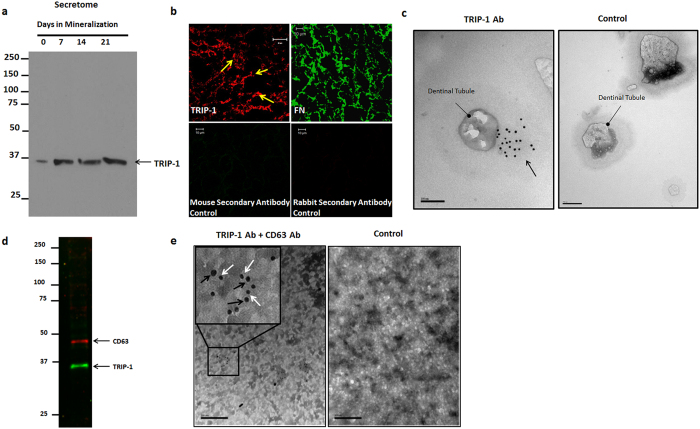
TRIP-1 is present in the ECM of preosteoblasts. (**2a**) Expression of TRIP-1 in the secretome of primary calvarial osteoblasts. (**2b**) Representative confocal micrographs of ECM isolated from MC3T3-E1 preosteoblasts immunostained for TRIP-1. Fibronectin was used as a positive control. The corresponding secondary antibodies were used as negative controls (**2c**) Representative unstained TEM images of dentin wafers showing the presence of immunogold labeled TRIP-1 (black arrows) in the dentin matrix around the dentinal tubules. (**d,e**): TRIP-1 is transported to ECM via exosomes. (**2d**) Western blot analysis of exosomes isolated from MC3T3-E1 cells show the presence of CD-63 an exosome marker and TRIP-1. (**2e**) Representative TEM image of solubilized exosomes showing the presence of immunogold labeled CD-63 (Black arrows, 20 nm) and TRIP-1 (White arrows, 10 nm). Inset shows the higher magnification image of the boxed area. Gold labeled anti-mouse (10 nm) and anti-rabbit (20 nm) secondary antibodies were used as control.

Western blot performed on the total protein from the secretome of primary calvarial cells under differentiation conditions showed increased expression through 21 days. This confirmed that TRIP-1 is a secretory protein synthesized by primary osteoblasts (Fig. 2a). To further confirm its extracellular localization, immunostaining was performed on the ECM derived from preosteoblast MC3T3-E1 cells. Results in Fig. 2b shows its presence in the matrix. Immunostaining with anti-fibronectin antibody served as the positive control.

We further confirmed the localization of TRIP-1 on the dentin extracellular matrix by using immunogold labeling technique using demineralized dentin wafer as the substrate. Figure 2c shows TRIP-1 labeled gold nanoparticles (10 nm) on the cross-sectional surface of dentinal tubules when compared with the control. (A low magnification image showing several dentinal tubules is shown in Supplementary Figure S2). Gold labeled (10 nm) secondary antibody alone served as the control. This data suggests that TRIP-1 can bind to the dense collagenous matrix of dentin.

### TRIP-1 is transported to ECM via Exosomes

TRIP-1 does not contain a classical signal peptide and so the presence of TRIP-1 in the ECM was intriguing. To identify a mechanism for transport of intracellular TRIP-1 to the ECM, we isolated exosomes from the secretome of MC3T3-E1 cells. Results in Fig. 2d show that TRIP-1 is secreted via the exosomal secretory pathway in CD63 positive exosomes confirmed by western blot analysis. Figure 2e shows representative TEM image of immunogold labeled exosomes showing the presence of CD63 (20 nm, Black arrows) and TRIP-1 (10 nm, White arrows). Low expression levels of TRIP-1 was observed in the exosome fraction from C2C12 cells while MDA-231 exosome fraction showed increased TRIP-1 expression (Supplementary Figure S3).

### Functional characterization of TRIP-1 in osteoblasts

**Figure 3:**
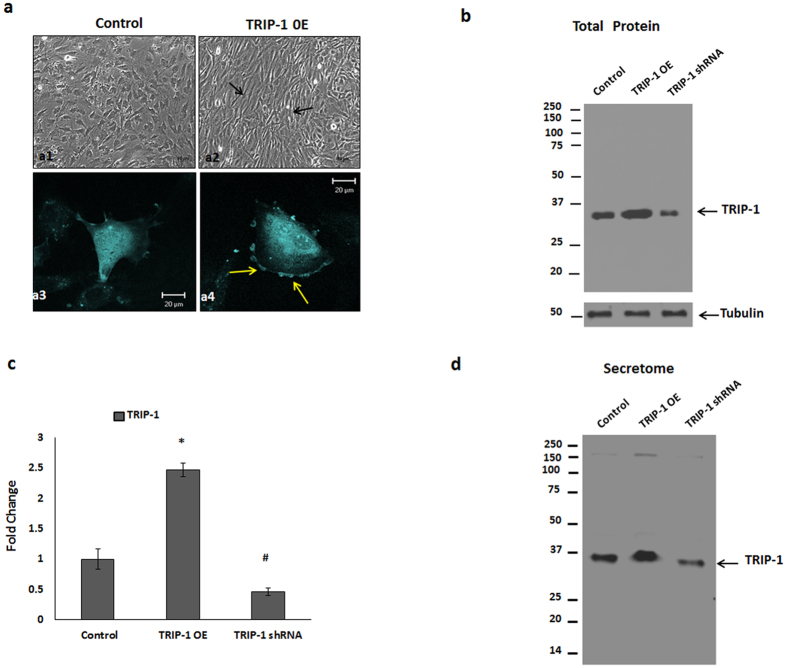
Overexpression and Silencing of TRIP-1 protein in MC3T3-E1 cells. CFP-TRIP-1 plasmid was used for establishing a stable cell line overexpressing TRIP-1. (**3a**) Light microscopy images of Control **(a1)** and TRIP-1 overexpressing cells **(a2).** Note the change in the cellular morphology (black arrows). (**3a3** and **3a4**) Confocal microscopy images of control and TRIP-1 overexpressing cells. Note the accumulation of TRIP-1 on the cell membrane as shown by yellow arrows (**a4**). Western blot analysis of total cell lysate **(3b)** and secretome **(3d)** from control, TRIP-1 overexpressed and TRIP-1 silenced cells shows the expression of TRIP-1. (**3c**) Quantitative analysis of TRIP-1 protein expression showing 2.5-fold increase in overexpressed cells and 70% reduction in expression in TRIP-1 silenced cells. Student’s *t*-test used to obtain statistical significance with respect to control. *, ^**#**^ Represents significance of p < 0.05.

To understand its function in osteoblasts, we overexpressed TRIP-1 in MC3T3-E1 cells and observed their morphology. Light microscopy analysis showed that with overexpression the cells changed their morphology from cobble-stone-like appearance (Fig. 3a–a1) to spindle-shaped cells, which were elongated, polarized and aligned themselves in straight parallel lines (Fig. 3a–a2). Confocal image of the cells showed accumulation of TRIP-1 on the plasma membrane (Fig. 3a–a4) when compared with the control (Fig. 3a–a3). Western blot analysis in Fig. 3b confirmed the overexpression and knocked down TRIP-1 protein levels in the total cell lysate and quantitative analysis showed a 2.5-fold increase and 70% reduction in TRIP-1 expression in overexpressed and shRNA treated cells respectively (Fig. 3c). Similar TRIP-1 expression pattern was observed in the secretome obtained from these cells. (Fig. 3d).

**Figure 4:**
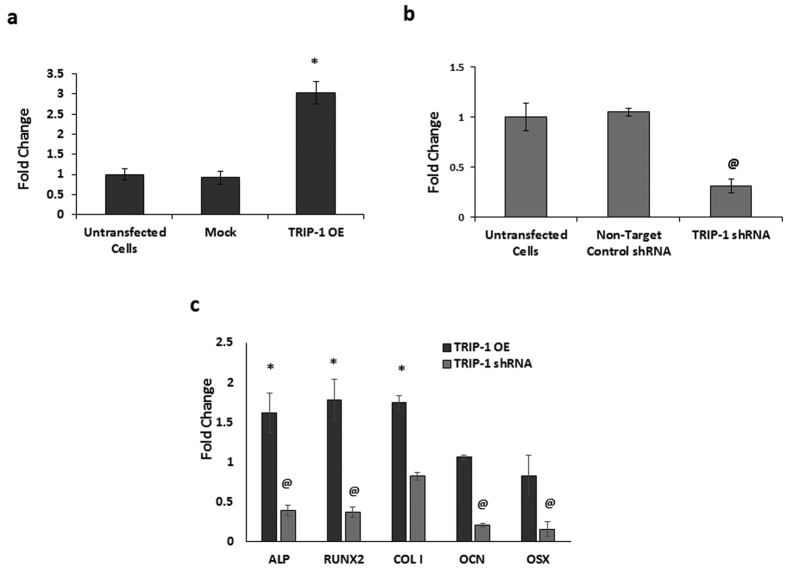
Overexpression and Silencing of TRIP-1 in MC3T3-E1 cells influences osteogenic differentiation. Real-Time PCR analysis depicting the expression levels of TRIP-1 in MC3T3-E1-TRIP-1 overexpressing cells **(4a)** and TRIP-1 knocked-down cells **(4b)**. A 3-fold increase in expression was observed in TRIP-1 OE cells when compared to untransfected cells. TRIP-1 expression decreased 70% in TRIP-1 knocked-down cells. No significant change in TRIP-1 expression was observed in mock vector transfected cells and non-target control shRNA treated cells. (**4c**) Real-Time PCR analysis showing osteogenic gene expression normalized to the control cells. Note increase in the expression levels of osteogenic differentiation markers such as ALP, Runx2, and Collagen type 1 in TRIP-1 OE cells. TRIP-1 silencing showed a downregulation in expression of ALP, Runx2 and OCN. Data are represented as mean fold change obtained from triplicate experiments with standard deviation as error. Student’s *t*-test was used to obtain statistical significance with respect to control. *, ^**@**^ Represents significance of p < 0.05.

RT-PCR quantitation showed a 3-fold increase of TRIP-1 in overexpressed cells (Fig. 4a) while efficient knockdown was observed in TRIP-1 shRNA cells (Fig. 4b). In each case, the expression levels was normalized to untransfected cells. Mock vector and non-target control shRNA were used as negative controls. We then analyzed the osteogenic gene expression profile in overexpressing and TRIP-1 knocked down MC3T3-E1 cells. Osteogenic gene expression and quantitation analysis showed upregulation of alkaline phosphatase (ALP), Runx2 Type I collagen, Osteocalcin (OCN) and Osterix (OSX). The expression of these genes were downregulated in TRIP-1 silenced cells (Fig. 4c). Protein level of Runx2 was confirmed by Western blot analysis in the control and transgenic cell lines (Supplementary Figure S4).

### TRIP-1 overexpression promotes mineralized matrix formation

**Figure 5:**
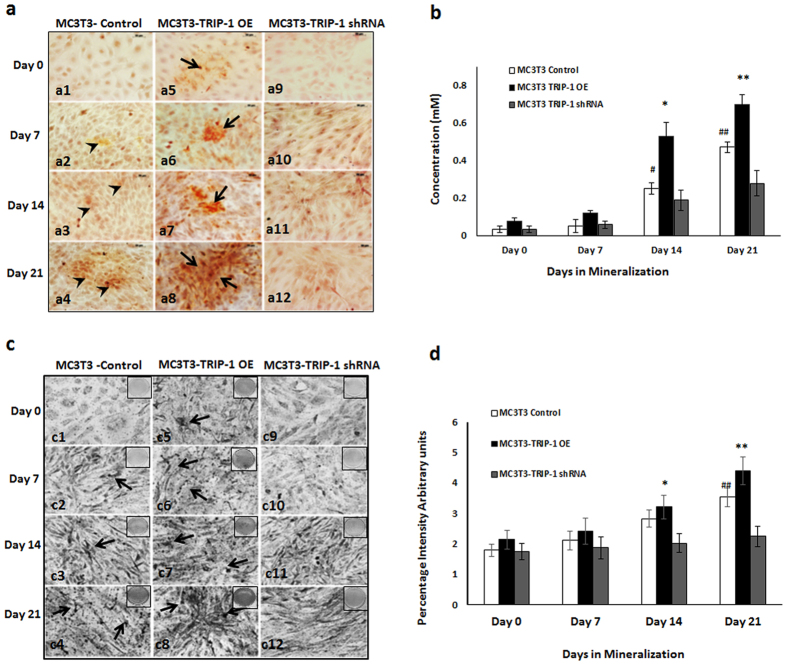
TRIP-1 influences matrix mineralization. MC3T3-E1 control cells, overexpressing TRIP-1 cells and shRNA mediated TRIP-1 knockdown cells were grown in osteogenic differentiation medium from 0 to 21 days. (**5a**) Representative Alizarin Red staining images demonstrate the presence of calcium in the matrix. Note the increased calcium deposition in TRIP-1 overexpressing cells from 0-21 days (Black arrows). Increased calcium deposition was also observed in control cells at 21 days albeit lesser than TRIP-1 overexpressed cells. Scale bars, 50 μm. (**5b**) Quantitative analysis of Alizarin Red staining. The cultures were destained and extracted using 10% acetic acid. Concentration determined measuring absorbance at 405 nm against Alizarin Red standard shows significant increase in 14 and 21-day TRIP-1 over expressing cells when compared to control cells. (**5c**) Representative images of von Kossa staining. Note the presence of intense black deposits indicating phosphate deposition in the matrix of cells overexpressing TRIP-1 (black arrows). (**5d**) Graph showing changes in the mineralization amounts in (**5c**), as percentage positive intensity units as analyzed by Image J. Students t test was used to calculate statistical significance. *, ^**#**^ Represents significance of p < 0.05.

The terminal stage in the osteogenic differentiation process is the formation of mineralized matrix. To assess TRIP-1’s function on matrix mineralization, Alizarin-red and von Kossa staining’s were performed on control, TRIP-1 overexpressing and TRIP-1 shRNA modified cells cultured under differentiation conditions up to 21 days. In Alizarin red staining, the matrix showed an increase in calcium deposition in control (black arrow heads, Fig. 5a–a1, a2, a3 and a4) and in TRIP-1 overexpressing cells (black arrows, Fig. 5a–a5, a6, a7 and a8.) for up to 21 days. In TRIP-1 silenced cells, reduced calcium deposition was observed (Fig. 5a–a9, a10, a11 and a12). These results were confirmed by quantitative analysis of the extracted alizarin red dye (Fig. 5b) and measuring the absorbance at 405 nm. von Kossa staining of TRIP-1 overexpressing cells (Fig. 5c–c5, c6, c7, c8) showed an increase in phosphate deposition up to 21 days when compared to TRIP-1 silenced cells (Fig. 5c–c9, c10, c11, c12). Quantitation by Image J analysis (Fig. 5d) showed that the observed increase in mineralization is statistically significant (*p < 0.05). In control MC3T3 cells (Fig. 5c–c1, c2, c3, c4) lesser amounts of phosphate was observed compared to TRIP-1 overexpressing cells. Supplementary Figure S5a shows the representative images of von Kossa staining on plates in which the cells were cultured up to 21 days. Increased ALP expression was observed in TRIP-1 overexpressing cells (Supplementary Figure S5b) grown up to 21 days in differentiation media when compared to control cells. A reduction in ALP activity was observed in TRIP-1 silenced cells.

### TRIP-1 binds to type I collagen

**Figure 6:**
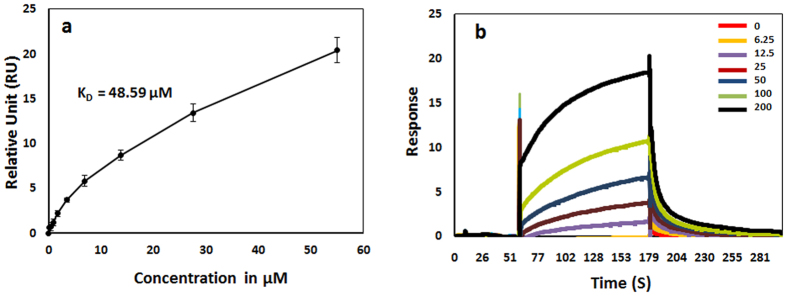
rTRIP-1 binds to Type 1 Collagen. (**6a**) Binding of rTRIP-1 to immobilized collagen I was analyzed by Surface plasmon resonance (SPR) spectroscopy. SPR data analyses show the steady state fits used to calculate the binding constant between rTRIP-1 and immobilized Type 1 Collagen (K_D_ = 48.59 μM). Values are the means from independent experiments that were performed in triplicates, and the error bars are the S.E.M. Figure 6b: SPR sensorgrams of a series of increasing concentrations of rTRIP-1 showing binding to immobilized type 1 Collagen.

To assess the role of TRIP-1 in collagen biomineralization, we performed binding assay between rTRIP-1 (Fig. S6) as the analyte and type 1 collagen as the ligand using Surface Plasmon Resonance (SPR). Steady state analysis (Fig. 6a) demonstrated that TRIP-1 bound to Collagen in a concentration-dependent and saturated manner with an apparent *K*_*D*_ of 48 μM. Figure 6b shows the sensorgrams of a series of increasing concentrations of rTRIP-1 flown on a Collagen 1-immobilized CM5 sensor surface clearly showing binding between TRIP-1 and type 1 Collagen.

### TRIP-1 promotes calcium phosphate deposition

**Figure 7:**
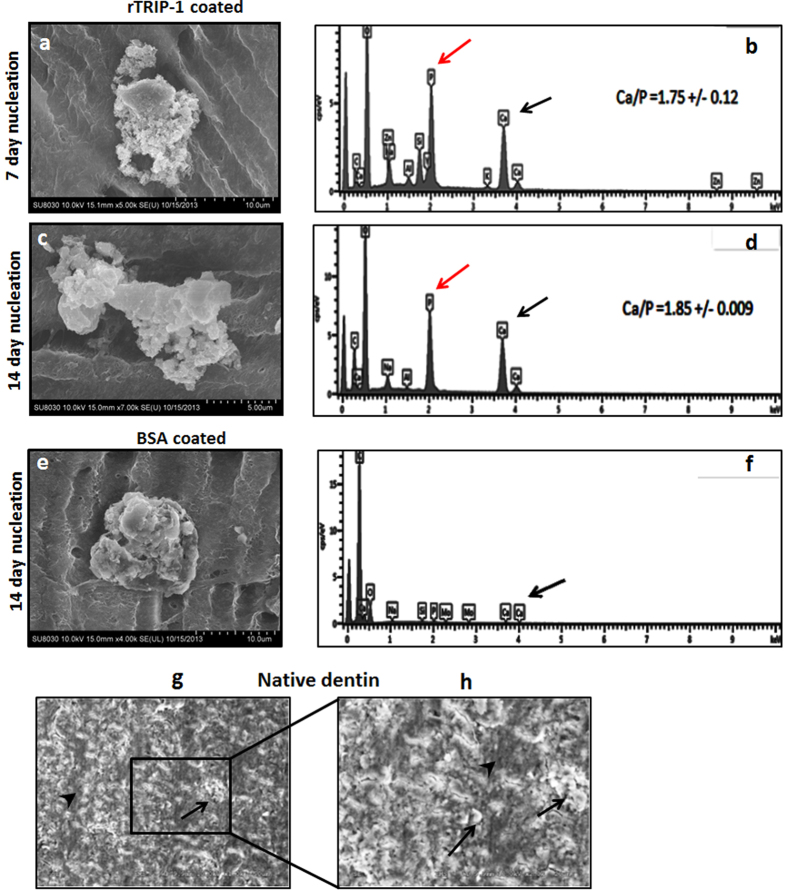
Scanning Electron microscopy analysis of the rTRIP-1 coated dentin wafer subjected to *in vitro* nucleation and the corresponding EDS analysis. (**7a** and **7c**) depict the representative SEM image of 100 μg rTRIP-1 coated demineralized and deproteinized dentin wafer subjected to *in-vitro* nucleation for 7 days **(7a)** and 14 days **(7c).** (**7b** and **7d**) represent the corresponding EDS analysis. (**7e**) Representative SEM image of 100 μg BSA coated on demineralized and deproteinized dentin wafer subjected to *in-vitro* nucleation for 14 days under physiological conditions**. (7f**) EDS spectra of BSA coated dentin wafers. (**7g and 7h**) Representative SEM images of native dentin. (**7h**) is the higher magnification of boxed area in (**7g**). Black arrows show mineral deposits and black arrowheads points to interfibrillar space.

To investigate the role of rTRIP-1 in biomineralization, we examined if rTRIP1 had the ability to nucleate calcium phosphate on the collagenous matrix of demineralized and deproteinized dentin wafer. SEM results showed that indeed rTRIP-1 could nucleate calcium phosphate polymorphs at 7 and 14 days respectively (Fig. 7a,c). EDX analysis of the mineral deposits showed the presence of calcium phosphate deposits and the Ca/P ratio was determined to be 1.75 and 1.85 at 7 & 14 days respectively (Fig. 7b,d). BSA coated dentin wafer also shows the presence of mineral crystals (Fig. 7e). EDX analysis (Fig. 7f) detected the presence of phosphate and calcium albeit in lesser amounts. SEM of native dentin wafer showing the mineral surface is shown in Fig. 7g,h.

**Figure 8:**
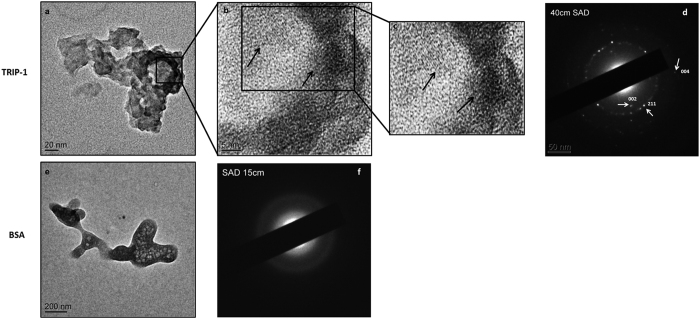
Transmission Electron microscopy analysis and corresponding selective area electron diffraction pattern(SAED) of rTRIP-1 nucleated mineral deposits. (**8a**) Representative unstained TEM image of mineral crystal nucleated by 20 μg of TRIP-1 on nickel grids subjected to mineralization for 1 h in the presence of 1 M Ca^2+^ and phosphate buffer. (**8b**) is the higher magnification of the boxed area in **8a**. Lattice image shows the presence of nanocrystalline arrays (black arrows) (**8c**) Digitally magnified images of 8b showing lattice fringes. (**8d**) Corresponding SAED image showing oriented crystals with strong reflections in the (002), (004) and (211) planes of hydroxyapatite. (**8e**) Representative TEM image of control protein (20 μg BSA) and corresponding SAED image (**8f**).

Transmission electron microscopy analysis of mineral nucleation initiated directly on EM grids showed that the mineral deposits on the rTRIP-1 coated surface was hydroxyapatite (Fig. 8a,b), based on the characteristic selected-area electron-diffraction (SAED) patterns with distinct (002), (004) and (211) reflections (Fig. 8d). The lattice fringes showed that the deposited mineral particles possessed long range crystallographic order (Fig. 8b,c). Figure 8e,f depicts the TEM image of BSA coated grid which was used as a control and its corresponding diffused diffraction pattern.

**Figure 9:**
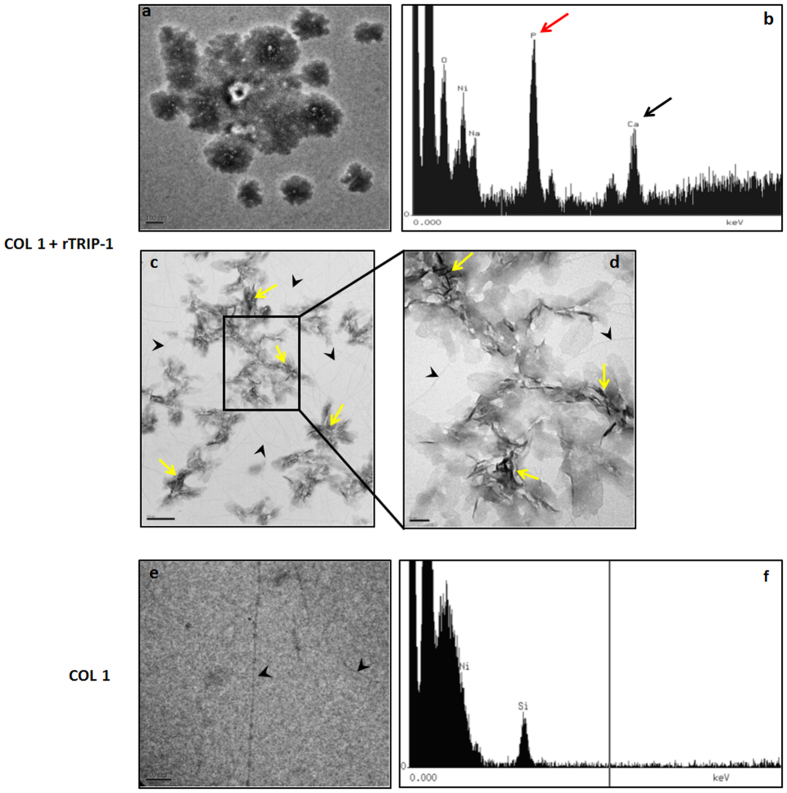
Transmission Electron microscopy analysis and corresponding EDS spectra of the mineral deposits in the presence of rTRIP-1 and Type I collagen. (**9a**) Representative unstained TEM image of mineral crystal nucleated by 20 μg of TRIP-1 on type I coated collagen nickel grids subjected to mineralization for 1 h in the presence of 1 M Ca^2+^ and phosphate buffer. (**9b**) Corresponding EDS analysis showing that the mineral deposits contain calcium and phosphate. (**9c & 9d**) Needle-shaped crystals (yellow arrows) emerge from the initial amorphous phase. Black arrowheads indicate type 1 collagen. (**9e & f**) Representative TEM image showing the absence of mineral deposits on the control grids coated with Type 1 collagen.

To determine whether TRIP-1 can bind collagen directly and initiate calcium phosphate nucleation, mineralization studies were performed directly on EM grids coated with collagen and rTRIP-1. TEM results showed that amorphous calcium phosphate deposits were initially observed (Fig. 9a,b) and then transformed to thin needle-like mineral crystals (Fig. 9c,d). The control type 1 collagen adsorbed grids did not show any mineral deposits (Fig. 9e,f).

### *In vivo* osteogenic differentiation potential of rTRIP-1 treated scaffolds and genetically modified cells

**Figure 10:**
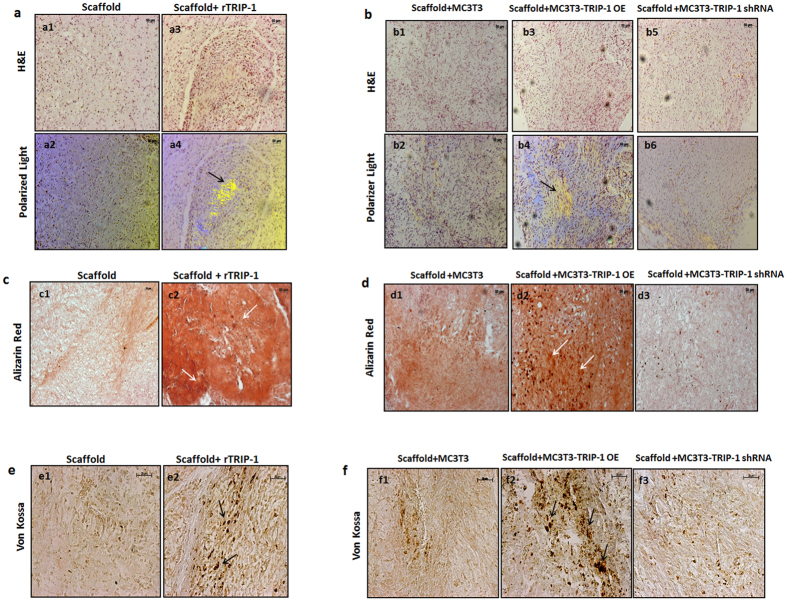
*In vitro* & *In vivo* histological evaluations and mineralization assay by using a subcutaneous transplantation model. (**10a**) Representative micrographs of H&E stained sections from control scaffold **(a1)** and rTRIP-1 treated scaffold **(a3)** after 4 weeks of subcutaneous implantation into immunodeficient mice. (**a2 and a4**) show collagen fibril orientation in control **(a2)** and rTRIP-1 treated scaffold **(a4)** implant sections as imaged by Polarized light. Note the increased deposition of oriented collagen fibers (Black arrows) in rTRIP-1 treated scaffold **(a4). (10b**) Representative micrographs of H&E stained sections from the MC3T3-E1 Control cells **(b1),** MC3T3-E1-TRIP-1 overexpressing cells **(b3)** and MC3T3-E1 TRIP-1 shRNA cells **(b5)** seeded on hydrogel scaffolds removed after 4 weeks of implantation. (**b2, b4 and b6**) show the collagen fibril orientation. Note the increased deposition of polarized collagen fibrils (Black arrows) in explant sections from scaffold treated with TRIP-1 overexpressing cells (**b4).** (**10c**) Representative micrographs of Alizarin Red staining of control scaffold **(c1)** and rTRIP-1 treated scaffold **(c2)** implant sections. White arrows show intense staining showing increased calcium deposition in scaffolds containing rTRIP-1 **(c2**). (**10d**) Representative Alizarin red staining images of sections from MC3T3-E1 control cells **(d1)** MC3T3-E1-TRIP-1 overexpressing cells **(d2)** and MC3T3-E1 TRIP-1 shRNA cells **(d3)** seeded on hydrogel scaffolds, after 4 weeks of implantation. Note the increase in calcium deposition (white arrows) with TRIP-1 overexpressing cells **(d2)** as compared with TRIP-1 knocked down cells **(d3).** (**10e**) Representative micrographs of von Kossa staining showing increase in staining in rTRIP-1 treated scaffolds (**10e2**) and in MC3T3-TRIP-1 OE seeded scaffolds (**10f2**).

*In vivo* function of TRIP-1 in biomineralization was assessed by subcutaneous implantation of 3D-scaffolds with or without rTRIP-1. Use of LZ (leucine zipper) hydrogel scaffolds for *in vivo* analysis has been published recently^18^. The explants with or without rTRIP-1 were harvested after 4 weeks. Histological examination of the tissues showed extensive cellularization (Fig. 10a–a3) and collagen deposition in the matrix as assessed by the birefringence of collagen under polarized light (Fig. 10a–a4) in the hydrogels containing rTRIP-1 when compared with the control (Fig. 10a–a1 & 10a–a2). Alizarin-Red and von-Kossa staining showed higher deposition of calcium and phosphate (Fig. 10c–c2 & 10e–e2) in hydrogels treated with rTRIP-1, when compared with the control (Fig. 10c–c1 & 10e–e1). Similar implantation experiments were performed with 3D-scaffolds containing control MC3T3-E1 and genetically modified MC3T3-E1 cells (TRIP-1 OE and TRIP-1 shRNA). Histological examination of the explants showed higher cell density, polarized collagen fibrils and calcified matrix deposition respectively (Fig. 10b–b3, b4 & 10d–d2) when compared with the control (Fig. 10b–b1, b2 & 10d-d1) and TRIP-1 silenced cells (Fig. 10b–b5, b6 & 10d–d3). Calcium and phosphate deposits were higher in the TRIP-1 overexpressing cells (Fig. 10d–d2 & 10f–f2) when compared with the knocked-down cells (Fig. 10d–d3 & 10f–f3) and control (Fig. 10d–d1 & 10f–f1).

### TRIP-1 promotes the expression of key osteogenic markers

**Figure 11:**
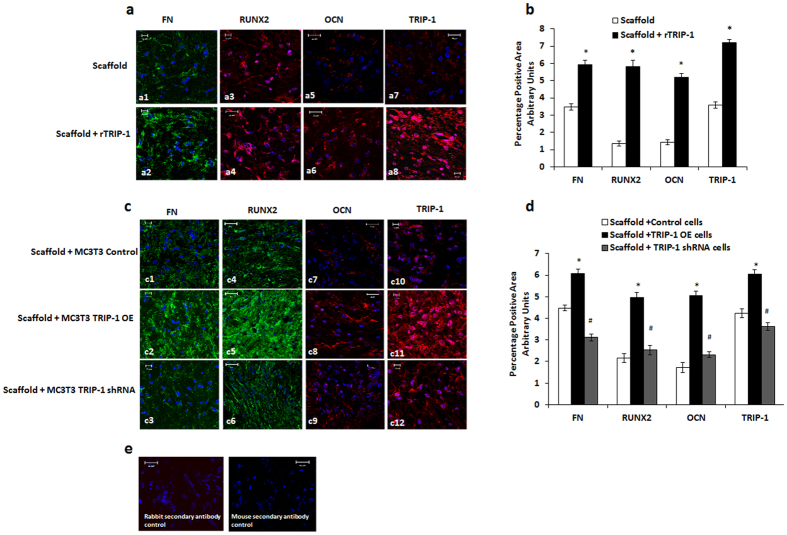
Expression of osteogenic markers in explant sections detected by immunolabeling. (**11a**) Representative confocal micrographs showing the immunohistochemical localization of fibronectin **(a1, a2)**, Runx2 **(a3, a4)**, OCN **(a5, a6)** and TRIP-1 **(a7, a8)** in control scaffold & scaffold +rTRIP-1 respectively. Nuclei were stained with DAPI. Note increased expression levels of these markers with rTRIP-1 treatment. Scale bar represents 20μm for all images. **11b:** Quantitation of the expression levels using Image J analysis and statistical significance obtained using Students t test. Significant increase in expression of FN, Runx2, OCN and TRIP-1 markers were observed in rTRIP-1 treated scaffolds (grey shaded box *p < 0.05) when compared to control scaffolds (white shaded box). (**11c**) Representative confocal micrographs showing immunohistochemical localization of Fibronectin **(c1, c2, c3)** Runx2 **(c4, c5, c6)**, OCN **(c7, c8, c9)** and TRIP-1 **(c10, c11, c12)** from explant sections of MC3T3-E1 control, MC3T3-E1-TRIP-1 overexpressing and MC3T3-E1 TRIP-1 shRNA cells seeded scaffolds respectively. (**11d**) Graph showing the percentage of positive area calculated using Image J. Statistical significance was calculated using Students t test. Expression of Fibronectin, Runx2, OCN and TRIP-1 markers show a significant increase in MC3T3–TRIP-1 OE cells seeded scaffolds (dark grey shaded box (*p < 0.05) when compared to control (white shaded box). Scaffolds preseeded with TRIP-1 knocked down cells show a significant reduction in expression (light grey shaded box, ^**#**^p < 0.05). (**11e**) Secondary antibody negative controls. Scale bar represents 10–20 μm.

To further elucidate whether TRIP-1 can influence osteogenesis at the cellular level *in-vivo*, the subcutaneously implanted hydrogel scaffolds pretreated with rTRIP-1 were subjected to immunocytochemical analysis. Results show an increase in the expression of fibronectin (FN), Runx2, Osteocalcin (OCN) and TRIP-1 (Fig. 11a–a2, a4, a6 and a8) when compared with the control scaffold (Fig. 11–a1, a3, a5 and a7).

Similarly, the explants from the genetically modified cells showed an increase in the expression profile of FN, Runx2, OCN and TRIP-1 (Fig. 11c–c2, c5, c8 and c11) respectively, when compared with the control (Fig. 11–c1, c4, c7 and c10) and the TRIP-1 silenced cells (Fig. 11c–c3, c6, c9 and c12).

Quantitation by Image J analysis (Fig. 11b,d) showed that the observed increase in mineralization is statistically significant (*p < 0.05) in both rTRIP-1 treated scaffolds and in scaffolds containing TRIP-1 overexpressing cells when compared to the respective controls. The decrease in mineralization observed with TRIP-1 silenced cells also showed statistical significance (^#^p < 0.05) when compared to TRIP-1 overexpressing cells.

## Discussion

The ECM of bone and dentin is diverse, with a variety of molecular components that bestow tissue-specific properties. Bone and dentin are biocomposites with remarkable hierarchical organization across all length scales from the atomic level to the macroscopic scale^19^,^20^,^21^. It is now well established that the self-assembly of type I collagen fibrils play a structural role in matrix mineralization^22^. In fact the superior mechanical properties of bone and dentin arise from the well-organized parallel arrangement of the calcium phosphate nanoparticles within the confined space of the self-assembled collagen matrix^23^. The functional collagen matrix can also interact with other NCPs to produce tissue-specific ECM conducive for mineralization. The NCPs in the bone ECM have been implicated to regulate crystal growth by initiating mineral nucleation on the collagen template or by directly binding to the crystallographic surfaces and inhibiting growth^24^,^25^,^26^. Identifying matrix proteins that can accelerate mineralization under physiological conditions is crucial for the biomechanical function of bone and dentin and could be used for tissue regeneration. Our group was the first to report that DMP1 an ECM protein in bone and dentin matrix can have intracellular functions^27^. In this study, we show that a predominantly intracellular protein TRIP-1, can be secreted out into the ECM and participate in matrix mineralization. Recently, several intracellular proteins that lack a signal peptide have been identified in the secretome of osteoblasts^28^.

In order for an intracellular protein such as TRIP-1 lacking a secretory signal peptide to function as a secreted protein, it is important to address the question as to how TRIP-1 moves from the ER into the cytoplasm and then to the extracellular matrix. In this study, we have demonstrated its localization in the exosomes secreted by MC3T3-E1 cells. Exosomes are small extracellular membrane vesicles secreted by several cell types and are enriched in CD63, a tetraspanin protein and is widely used as a marker protein for exosomes. Exosomes have been reported to carry proteins, mRNA, microRNAs and to facilitate transfer of genetic information between cells^29^ Recent studies have shown that exosomes and matrix vesicles are homologous structures^30^ and exosomes anchor to the extracellular matrix and adopt the morphological appearance and functional activities of matrix vesicles^30^. Earlier studies have documented that matrix vesicles serve as initial sites for mineral formation in vertebrate mineralizing tissues^31^. The presence of TRIP-1 in the exosomes, suggest that this could be the mode of transport by which the protein is packaged and released to the self-assembled collagen matrix in the ECM. Thus, TRIP-1 is released to the ECM by non-classical secretory mechanisms where it can orchestrate the formation of a mineralized matrix.

Mineralized matrix formation is a cellular event and is orchestrated by the synthesis and secretion of several proteins, complex interactions between ECM components, cell surfaces, growth factors, morphogens and cytokines. Gene expression analysis showed that overexpression of TRIP-1 accelerated cellular differentiation by synthesizing osteogenic genes such as alkaline phosphatase, Runx2 and OCN. An increase in the expression of osteogenic markers directly correlated well with the differentiation activity at the cellular level. Silencing of TRIP-1, resulted in significant reduction of osteogenic gene expression and supports the finding that TRIP-1 may promote osteoblast differentiation^1^. Cells overexpressing TRIP-1 showed increased calcium and phosphate deposition in cultures at 14 and 21 days. However, TRIP-1 silenced cells showed a reduction in matrix mineralization. These findings imply that TRIP-1 can aid in osteoblast differentiation and mineralized matrix formation.

As the function of TRIP-1 in the ECM is unknown, therefore, we characterized its role in mineralization via its ability to bind calcium and initiate the nucleation process under physiological and high Ca^2+^ and Pi concentrations. In attempting to mimic the dense three-dimensional collagen environment in mineralized matrices of bone and dentin, we examined if rTRIP-1 coated demineralized and deproteinized dentin wafers could promote calcium phosphate mineral deposition and growth. Demineralized dentin wafer mimic’s the natural arrangement of type I collagen and indicate physiological relevance. SEM results showed mineralized dentin matrix comparable to native dentin. TEM micrographs confirmed the mineralization of the demineralized dentin wafer coated with TRIP-1, as the collagen banding pattern due to mineral deposition was clearly evident in unstained images (Supplementary Figure 7). Interactions of noncollagenous proteins with collagen fibrils are necessary to promote site specific mineralization as nucleation occurs initially in the gap regions within the collagen fibril. Mineralized collagen fibrils represent the structure at the lowest level of hierarchy used by nature in building mineralized tissues.

Investigation of the influence of TRIP-1 with calcium phosphate mineral by TEM showed the nanoscale details of the evolution of needle-shaped mineral deposits. The growing nanoclusters appeared in various sizes and the diffraction pattern confirmed the amorphous nature. However, with time crystalline deposits were obtained as assessed by the long range crystallographic order in the mineral deposits. The mineral deposits had sufficient crystallinity to obtain a diffraction pattern, while the control BSA showed a diffused ring pattern.

The possibility of rTRIP-1 to nucleate calcium and phosphate led us to investigate if rTRIP-1 can directly bind to type I collagen. Using surface plasmon resonance (SPR) analysis, we found that rTRIP-1 bound to immobilized type I collagen in a dose-dependent manner with a *K*_*D,*_ a measure of the affinity between the two molecules of 48.59 μM and with fast association and dissociation rates. This suggests that TRIP-1 binds with Type I Collagen and this binding affinity is relatively weaker than the binding of DMP1 and collagen^32^. Immunogold labeling on monomeric type I collagen showed TRIP-1 binding to collagen aggregates (Supplementary Figure 8). Based on these observations, it is plausible to conclude that TRIP-1 secreted to the ECM by exosomes could bind collagen and promote matrix mineralization.

In this study we have presented exciting observations that TRIP-1 is transported to the ECM via exosomes. In the ECM TRIP-1 binds to type I collagen and promotes matrix mineralization. Importantly, we also show that knockdown of TRIP-1 negatively impacts osteogenic differentiation and matrix mineralization. Thus, our study provides compelling evidence for a regulatory role for TRIP-1 in the calcification process.

## Conclusions

Our findings bear exciting implications in biomineralization. In calvarial osteoblasts, we have shown that TRIP-1 predominantly an intracellular protein is localized in the extracellular matrix. This finding poses a conceptual problem on how TRIP-1 can be routed to the ECM without a secretory signal. We have determined that TRIP-1 is transported out to the matrix in exosomes. There is emerging evidence that exosomes might transport mineral and proteins to the ECM of vertebrate mineralizing tissues. Further, TRIP-1 expression increases with matrix production and mineralization. In the matrix, TRIP-1 can bind specifically to type I collagen and initiate the calcium phosphate nucleation process. Using genetically modified cells containing TRIP-1 overexpressing or silenced cells we showed that TRIP-1 can promote preosteoblast differentiation and produce mineralized matrix formation thus confirming its ever expanding functional versatility. Identifying the role of new proteins in the mineralized matrix is essential for designing tailored materials with tunable properties for bone repair and regeneration.

## Methods

### Cell Culture

MC3T3-E1, mouse preosteoblast cell line obtained from ATCC and primary calvarial cells isolated from 3 day old wild type mouse pups (n = 5) were used in this study. The MC3T3-E1 cells were cultured in DMEM/F12 (Corning, Corning, NY) supplemented with 10% FBS and 1% antibiotics. Primary mouse calvarial osteoblasts were cultured in alpha minimum essential medium (Corning, Corning, NY) supplemented with 20% fetal bovine serum and 1% antimycotic antibiotic. For *in vitro* mineralization experiments, the normal growth medium was supplemented with 10 mM β-glycerophosphate, 100 mg/ml ascorbic acid (Sigma Chemical Company, St. Louis, MO) and 10 nM dexamethasone (MP Biomedicals, Santa Ana, CA).

### Expression and purification of recombinant TRIP-1

Recombinant TRIP-1 protein was expressed in bacteria using pQE-30 plasmid (Qiagen Inc, Valencia, CA) system. Briefly, 973-bp fragment corresponding to the coding region of TRIP-1 was cloned into Sph I/Sal I restriction sites in pQE-30 vector. This plasmid was transformed into *E. coli* bacteria. Briefly, a single bacterial colony was inoculated in 500 mL LB broth with 100 μg/mL ampicillin and 30 μg/mL kanamycin and incubated overnight in a 37 °C shaker The culture was then transferred into larger 2 L LB broth culture with 100 μg/mL ampicillin and 30 μg/mL kanamycin and incubated in a 37 °C shaker until OD 0.6–0.8 was reached. The protein was expressed by the addition of 1 mM isopropyl β-D-1-thiogalactopyranoside (IPTG) (Thermo Fisher scientific, Waltham, MA) to the culture and grown for 5 hours. The cells were then pelleted and stored at −80 °C. Protein purification was carried out using Ni-NTA Superflow resin (Qiagen) under native conditions following manufacturer’s protocol. The elution buffer with TRIP-1 recombinant protein was dialyzed, lyophilized and stored at −20 °C until use. The purified protein was then run on SDS-PAGE gel and stained with Coomassie blue to determine protein quality.

### Overexpression and Silencing of TRIP-1

For stable overexpression, rat TRIP-1 cDNA was PCR-amplified and cloned into HindIII and ApaI sites of pECFP vector (Clontech Laboratories, Mountain View, CA). Stable transfections with TRIP-1-CFP plasmid were performed on MC3T3-E1 cells as published earlier^17^. Cells mock transfected with empty vector served as the control. For knock down studies, mission shRNA clones for TRIP-1 and mission non-target shRNA control was purchased as glycerol stocks from Sigma Chemical Company (St. Louis, MO. Plasmid DNA was purified and used to generate a stable cell line with TRIP-1 gene knocked down. Cells transfected with scrambled shRNA was used as the control.

### Exosome isolation from secretome

Media was collected from confluent MC3T3-E1 cells and centrifuged at 400 g at 4 °C for 5 min to remove debris. The cleared supernatants were processed using Exosome extraction kit (Life Technologies, Carlsbad, CA) according to the manufacturer’s protocol. The final pellet was suspended in 200 μl of PBS and processed for western blot analysis and for TEM.

### Protein Isolation and Western Blotting

Total proteins were extracted from primary calvarial osteoblast cells using M-Per reagent (Pierce, Rockford, IL). Thirty μg of the total proteins were resolved on a 10% SDS-polyacrylamide gel under reducing conditions. After electrophoresis, the proteins were electro-transferred onto nitrocellulose membrane (Bio-Rad Laboratories, Hercules, CA), blocked with 5% nonfat milk, probed with rabbit polyclonal Eif3β (1/500, Santacruz Biotechnology) and mouse monoclonal RUNX2 antibody (1/1000, Santacruz Biotechnology). Tubulin was used as control to ensure equal loading of proteins. Blots were then incubated with HRP-conjugated goat anti-mouse IgG (Sigma Chemical Company, St. Louis, MO). After washing three times with PBS containing 0.05% Tween 20 and once with PBS, the bands were visualized by the lightening chemiluminescence reagent (Pierce, Rockford, IL).

To obtain secreted proteins, equal number of mouse calvarial preosteobalst cells were grown in 100 mm tissue culture dishes in presence of differentiation media for 7, 14 and 21 days while 0 day served as the control. The cells were grown to complete confluence, and the medium was changed to serum-free regular media or serum free differentiation media 48 hr before the desired time point. At the end of the time point, the medium was collected, and the cells were trypsinized and counted to ensure that the cell number did not vary significantly between the time points. The collected media was dialyzed against deionized water, lyophilized and reconstituted in 500 μl of PBS. 15 μl of the reconstituted protein sample obtained from media collected from a single plate at each time point was resolved on SDS-PAGE and immunoblotting was performed as described previously.

Western blotting analysis of exosomes was carried out using Odyssey (^®^) Infrared Imaging system (Li-Cor Biosciences, Lincoln, NE). Briefly, 20 μl of the resuspended exosome solution was resolved on 10% SDS polyacrylamide gel and transferred to nitrocellulose membrane. After blocking with Odyssey blocking buffer (Li-Cor Biosciences), the membrane was incubated with rabbit polyclonal CD63 (1/500) and mouse monoclonal EIF3β (1/500) primary antibodies (Santacruz Biotechnology, Dallas, TX) followed by incubation in infrared labeled goat anti-rabbit IRDye 680 and goat anti-mouse IRDye 800 (Li-Cor Biosciences) secondary antibodies. The bound complex was detected using the Odyssey Infrared Imaging System (Li-Cor Biosciences).

### Real-time PCR

Control MC3T3-E1 cells, MC3T3-E1 overexpressing TRIP-1 and MC3T3 TRIP-1 shRNA cells were cultured to confluence. Total RNA was isolated using the Qiagen RNA isolation kit as per the manufacturer’s instructions. First strand synthesis was performed using First strand cDNA synthesis kit (Qiagen). The generated cDNA was subjected to gene specific quantitative PCR amplification. mRNA levels encoding for TRIP-1, RUNX2, ALP, OCN, GAPDH and HPRT were obtained using primers synthesized (Integrated DNA Technologies, Coralville, IA) from published sequences (Table 1). Fold change was calculated using –ΔΔCT method). Statistical significance between the control and TRIP-1 overexpressing cells were calculated using student’s t-test. All experiments were carried out in triplicates.

### Characterization of the mineralized matrices by von Kossa, Alizarin Red S and ALP staining

The MC3T3-E1 control, MC3T3-E1 overexpressing TRIP-1 and cells stably transfected with TRIP-1 shRNA were cultured in osteogenic differentiation media for 0, 7, 14 and 21 days. At each time point the cells were washed with distilled water and fixed in 10% neutral formalin. For von Kossa staining, the cells were washed with deionized water and treated with 1% AgNO3 for 1hr, washed again with distilled water and treated with 2.5% sodium thiosulphate for 5 min. For alizarin red staining, the cells were washed with deionized water and stained for 10 minutes with 2% Alizarin Red S solution pH 4.2, washed again with deionized water and dehydrated in graded ethanol solutions. The cells were imaged using a Zeiss Axio Observer D1 microscope equipped with Axiovision Imaging software. Extraction of Alizarin Red staining and quantitative analysis was carried out as per the published protocol^33^. Quantitation of von Kossa staining was done using Image J analysis. For ALP staining, the cells were fixed in ice cold methanol for 10 min and incubated with equal volumes of NBT/BCIP reagent (Bio-Rad Laboratories) for 30 min. All experiments were carried out in triplicates.

### Immunogoldcytochemistry of exosomes and dentin matrix

20 μl of the resuspended exosome preparation was added on 300 mesh formvar coated nickel grids. The grids were fixed in 10% neutral formalin, permeablized with PBS containing 1% Triton-X 100 for 30 minutes and blocked with 3% BSA for 1 hour at room temperature. Following blocking, the grids containing exosomes were incubated with rabbit polyclonal CD63 (1/500) and mouse monoclonal EIF3β (1/500) primary antibodies (Santacruz Biotechnology) overnight. The grids were washed with PBS containing 1% tween, followed by incubation with gold conjugated anti-rabbit IgG (20 nm) and anti-mouse IgG (10 nm). The grids were extensively washed in PBST, rinsed in water and allowed to air dry. The exosome containing grids incubated with only the gold conjugated anti-mouse and anti-rabbit IgG served as controls. All the grids were stained with uranyl acetate and analyzed by TEM.

The demineralized dentin wafer tissues (kind gift from Dr. Bedran-Russo, Department of Restorative Density, UIC) were treated with trypsin to remove the noncollagenous proteins as published earlier^34^,^35^. The wafers were dehydrated in a series of ethanol from 30 to 100%, trimmed, embedded in epoxy resin and ultrathin sections (70 nm) were placed on 300 mesh formvar carbon coated nickel grids. Grid-mounted tissue sections were processed for colloidal-gold immunocytochemistry by incubating the sections with mouse primary antibody against EIF3β (TRIP-1) (Santacruz Biotechnology), after which immunolabeling was visualized by incubation with anti-mouse IgG colloidal gold complex (10 nm gold particles). For controls, the sections were incubated with 10 nm gold conjugated antimouse IgG alone. All the grids were analyzed by TEM.

### Surface Plasmon Resonance

Surface plasmon resonance (SPR) analyses were performed using a BIAcore T200 instrument (GE Healthcare, Marlboro, MA, USA). Recombinant rat tail Type 1 Collagen (1 mg/ml, Bbiosciences) was immobilized on a CM5 sensor chip (Series S Sensor Chip CM5; GE Healthcare) using standard amine coupling. In short, flow channels were activated by 1-ethyl-3- (3-dimethylaminopropyl) carbodiimide hydrocholoride (EDC)/N-hydroxy succinimide (NHS) mixture, and protein was immobilized followed by ethanolamine blocking of unoccupied surface area at 25 °C. Blank immobilization was performed on flow channel 1 as a control. Immobilization levels of flow channel 2 was 2622 response units (RU). Recombinant TRIP-1 binding was measured at a flow rate of 20 μL/min in running buffer PBS-P (0.5% Surfactant P20 [Tween 20], pH 7.4) containing varied concentrations (0–200 μg at 2-fold dilution) of analyte protein. Data were normalized with blank (ethanolamine) RU values. Binding affinities were determined from steady state analysis of maximum steady state response.

### *In-vitro* nucleation

*In-vitro* nucleation was performed in the electrophoresis chamber as published earlier^36^. Three types of techniques were used. In the first technique, demineralized dentin wafers were treated with trypsin to remove the noncollagenous proteins as published earlier^37^,^38^. The processed dentin wafers were coated with 100 μg of rTRIP-1 and used in *in-vitro* nucleation experiments Briefly, rTRIP-1 coated dentin wafers were placed into a channel connecting two halves of an electrolytic cell, with one compartment containing calcium buffer (165 mM NaCl, 10 mM HEPES, 2.5 mMCaCl_2_, pH 7.4) and the other phosphate buffer (165 mM NaCl, 10 mM HEPES, 1 mMKH_2_PO_4_, pH 7.4). A small electric current of 1 mA was passed through the system to facilitate even distribution of the ions on the dentin wafers coated with the proteins. The buffers were changed regularly to maintain a constant pH and were tested periodically. 100 μg BSA coated dentin wafers and dentin wafers with no protein coated served as the control.

The second and third technique involved using formvar carbon coated nickel grids. For the second technique, Carbon-formvar-coated nickel grids (Ted Pella) were incubated with 20 μg of rTRIP-1 protein solution for 1 h at room temperature. For control, BSA coated grid was used. The third was similar to the above technique, except that solubilized type I collagen was reconstituted on formvar coated nickel grids before incubating with rTRIP-1 protein. Type 1 collagen adsorbed grids (no protein coated) served as the control. The grids were then washed with water and suspended in 1 M CaCl_2_ (pH 7.4) for 1 h followed by incubation in 1 M KH_2_PO4 (pH 7.4). The grids were extensively washed with water and allowed to air dry. The samples were examined with a Transmission electron microscope.

### Electron microscopy

The gold labeled exosome grids and gold labeled dentin wafer were imaged in JEM JEOL 1220 Electron Microscope and digital images obtained using Erlangshen ESW 1000w 785 camera.

For dentin wafer substrates, after the specific time points the samples were washed and dehydrated by passing through a series of graded ethanol solutions of 30%, 50%, 90% and 100% for 10 min each. The samples were finally dehydrated by immersing them in a solution of hexamethyldisilazane (HMDS) for 10 min followed by air-drying inside a tissue-culture hood. Samples were imaged using a JOEL JSM 6320F field emission scanning electron microscope.

The formvar carbon coated nickel grids were imaged and analyzed on TEM for selected area electron diffraction (SAED) and atomic resolution imaging using a TEM (JEM-3010, JEOL) at 300 kV.

### *In-vivo* assay to determine the mineralization potential of TRIP-1

Subcutaneous implants were performed on 1 month old male immunocompromised nude mice purchased from Charles River Laboratories. Leucine-zipper hydrogel (7%) synthesized in our lab was used as a scaffold^18^. Two groups were used in this study. The first group consisted of hydrogel scaffold pretreated with rTRIP-1 protein while the hydrogel with no treatment served as control. In the second group control MC3T3-E1 cells, MC3T3-E1 overexpressing TRIP-1 and MC3T3-E1 TRIP-1 shRNA cells were seeded at a density of 2 × 10^6^ cells/scaffold in triplicates. The cell seeded scaffolds were cultured *in vitro* for 48 h and were then implanted subcutaneously on the back of athymic nude mice (Charles River Laboratories). Four weeks post implantation, the animals were sacrificed and the scaffolds were retrieved, fixed in 4% neutral buffered formalin, embedded and sectioned into 5 μm thick sections for histological evaluation. Three tissue sections from each of the group were analyzed. Three fields per section was imaged and analyzed. All animal experiments were performed as per the protocol approved by the UIC animal care committee (Assurance number A-3460-01). All experiments were carried out in accordance with relevant guidelines and regulations.

### Histology and Immunohistochemistry

Sections were deparaffinized in xylene, hydrated in graded ethanol solutions, and hematoxylin and eosin (H&E) staining were performed as per published protocols^37^,^38^. Alizarin red staining to visualize calcium deposition was performed as per standard procedures. Immunohistochemistry using peroxidase conjugated secondary antibodies or fluorescent probes according to published protocols. The following antibodies were used, rabbit anti-fibronectin (FN) antibody (1/100, Sigma), mouse-runt-related transcription factor 2 (RUNX2) antibody (1/100, Abcam), mouse osteocalcin (OCN) antibody (1/1000, Abcam) and mouse EIF3β (TRIP-1) antibody (1/500 Santacruz Biotechnology). All fluorescently stained sections were imaged at the University of Illinois at Chicago Research Resource Center core imaging facility. Imaging was performed using a Zeiss LSM 710 confocal microscope equipped with Zen image analysis software. All comparative fluorescence images were obtained using the same imaging conditions.

### Statistical analyses

Data are presented as the mean ± standard deviation of at least 3 independent experiments. Statistical analysis of the data was calculated using the Student’s *t* test. *P* < 0.05 was considered significant.

## Additional Information

**How to cite this article**: Ramachandran, A. *et al.* TGF beta receptor II interacting protein-1, an intracellular protein has an extracellular role as a modulator of matrix mineralization. *Sci. Rep.* **6**, 37885; doi: 10.1038/srep37885 (2016).

**Publisher's note:** Springer Nature remains neutral with regard to jurisdictional claims in published maps and institutional affiliations.

## Supplementary Material

Supplementary Information

## Figures and Tables

**Table 1:** Real Time PCR Primer Sequences.

Gene	Forward Primer (5′ → 3′)	Reverse Primer (5′ → 3′)
ALP	GTGCCAGAGAAAGAGAGAGA	TTTCAGGGCATTTTTCAAGGT
OCN	CTCCTGAGAGTCTGACAAAGCCTT	GCTGTGACATCCATTACTTGC
RUNX2	CCTGAACTCTGCACCAAGTC	GAGGTGGCAGTGTCATCATC
TRIP-1	GCAGATGGGGTATCAGTACT	ACGTTCACCAACACCTCTCC
GAPDH	ACCACAGTCCATGCCATCAC	CACCACCCTGTTGCTGTAGCC
HPRT	TCAGTCAACGGGGGACATAAA	GGGGCTCTACTGCTTAACCAG

## References

[b1] Metz-Estrella D., Jonason J. H., Sheu T. J., Mroczek-Johnston R. M. & Puzas J. E. TRIP-1: a regulator of osteoblast function. J Bone Miner Res. 27, 1576–1584 (2012). 22460930 10.1002/jbmr.1611 PMC3377841

[b2] Choy L. & Derynck R. The type II transforming growth factor (TGF)-beta receptor-interacting protein TRIP-1 acts as a modulator of the TGF-beta response. J. Biol. Chem. 273, 31455–31462 (1998). 9813058 10.1074/jbc.273.47.31455

[b3] Sheu T. J. *et al.* A phage display technique identifies a novel regulator of cell differentiation. J Biol. Chem. 278 **(1)**, 438–443 (2003). 12403789 10.1074/jbc.M208292200

[b4] Ahlemann M. *et al.* Carcinoma-associated eIF3i overexpression facilitates mTOR-dependent growth transformation. Mol. Carcinog. 45, 957–967 (2006). 16929481 10.1002/mc.20269

[b5] Rauch J. *et al.* Allogenic antibody-mediated identification of head and neck cancer antigens. Biochem Biophys Res Commun. 323 **(1)**, 156–162 (2004). 15351715 10.1016/j.bbrc.2004.08.071

[b6] Huang J. S. *et al.* Diverse cellular transformation capability of overexpressed genes in human hepatocellular carcinoma. Biochem Biophys Res Commun. 315 **(4)**, 950–958 (2004). 14985104 10.1016/j.bbrc.2004.01.151

[b7] Yuan Y. *et al.* The Translation Initiation Factor eIF3i Up-regulates Vascular Endothelial Growth Factor A, Accelerates Cell Proliferation, and Promotes Angiogenesis in Embryonic Development and Tumorigenesis. J. Biol. Chem. 289, 28310–28323 (2014). 25147179 10.1074/jbc.M114.571356 PMC4192485

[b8] Wang C. *et al.* Clusterin facilitates metastasis by EIF3I/Akt/MMP13 signaling in hepatocellular carcinoma. Oncotarget. 6 **(5)**, 2903–2916 (2015). 25609201 10.18632/oncotarget.3093 PMC4413626

[b9] Asano K., Kinzy T. G., Merrick W. C. & Hershey J. W. Conservation and diversity of eukaryotic translation initiation factor eIF3. J. Biol. Chem. 272, 1101–1109 (1997). 8995409 10.1074/jbc.272.2.1101

[b10] Marchione R., Leibovitch S. A. & Lenormand J. L. The Translational Factor eIF3f: The Ambivalent eIF3 Subunit. Cellular and Molecular Life Sciences 70 **(19)**, 3603–3616 (2013). 23354061 10.1007/s00018-013-1263-y PMC3771369

[b11] Masutani M., Sonenberg N., Yokoyama S. & Imataka H. Reconstitution reveals the functional core of mammalian eIF3. The EMBO Journal 26, 3373–3383 (2007). 17581632 10.1038/sj.emboj.7601765 PMC1933396

[b12] Mullen C. A., Haugh M. G., Schaffler M. B., Majeska R. J. & McNamara L. M. Osteocyte differentiation is regulated by extracellular matrix stiffness and intercellular separation. J Mech Behav Biomed Mater. 28, 183–194 (2012). 10.1016/j.jmbbm.2013.06.013 PMC5776008 23994943

[b13] Opsahl V. S. *et al.* Tooth dentin defects reflect genetic disorders affecting bone mineralization. Bone. 50, 989–997 (2012). 22296718 10.1016/j.bone.2012.01.010 PMC3345892

[b14] Neary M. T. *et al.* Contrasts between organic participation in apatite biomineralization in brachiopod shell and vertebrate bone identified by nuclear magnetic resonance spectroscopy. J R Soc Interface. 8, 282–288 (2012). 10.1098/rsif.2010.0238 PMC3033023 20610423

[b15] Ehrlich H., Koutsoukos P. G., Demadis K. D. & Pokrovsky O. S. Principles of demineralization: modern strategies for the isolation of organic frameworks. Part II. Decalcification. Micron. 40, 169–193 (2009). 18804381 10.1016/j.micron.2008.06.004

[b16] Veis A. & Dorvee J. R. Biomineralization mechanisms: a new paradigm for crystal nucleation in organic matrices. Calcif Tissue Int. 93, 307–315 (2012). 23241924 10.1007/s00223-012-9678-2 PMC3726565

[b17] Ramachandran A., Ravindran S. & George A. Localization of transforming growth factor beta receptor II interacting protein-1 in bone and teeth: implications in matrix mineralization. J Histochem Cytochem. 60, 323–337 (2012). 22260994 10.1369/0022155412436879 PMC3351241

[b18] Huang C. C., Ravindran S., Yin Z. & George A. 3-D self-assembling leucine zipper hydrogel with tunable properties for tissue engineering. Biomaterials. 35 **(20)**, 5316–5326 (2014). 24713184 10.1016/j.biomaterials.2014.03.035 PMC4020426

[b19] Reznikov N., Shahar R. & Weiner S. Three-dimensional structure of human lamellar bone: the presence of two different materials and new insights into the hierarchical organization. Bone. 59, 93–104 (2014). 24211799 10.1016/j.bone.2013.10.023

[b20] Tuer A. E. *et al.* Hierarchical model of fibrillar collagen organization for interpreting the second-order susceptibility tensors in biological tissue. Biophys. J. 103, 2093–2105 (2012). 23200043 10.1016/j.bpj.2012.10.019 PMC3512050

[b21] Wang Y. *et al.* The predominant role of collagen in the nucleation, growth, structure and orientation of bone apatite. Nat. Mat. 11, 724–733 (2012). 10.1038/nmat3362 22751179

[b22] Nudelman F. *et al.* The role of collagen in bone apatite formation in the presence of hydroxyapatite nucleation inhibitors. Nat. Mat. 9, 1004–1009 (2012). 10.1038/nmat2875 PMC3084378 20972429

[b23] Nudelman F., Lausch A. J., Sommerdijk N. & Sone E. D. *In vitro* models of collagen biomineralization. J. Struct. Biol. 183, 258–269 (2012). 10.1016/j.jsb.2013.04.003 23597833

[b24] George A. & Veis A. Phosphorylated proteins and control over apatite nucleation, crystal growth, and inhibition. Chem. Rev. 108, 4670–4693 (2008). 18831570 10.1021/cr0782729 PMC2748976

[b25] Hunter G. K., O’Young J., Grohe B., Karttunen M. & Goldberg H. A. The Flexible Polyelectrolyte Hypothesis of Protein−Biomineral Interaction. Langmuir. 26 **(24)**, 18639–18646 (2010). 20527831 10.1021/la100401r

[b26] Hoac B., Kiffer-Moreira T., Millán J. L. & McKee M. D. Polyphosphates inhibit extracellular matrix mineralization in MC3T3-E1 osteoblast cultures. Bone. 53 **(2)**, 478–486 (2013). 23337041 10.1016/j.bone.2013.01.020 PMC3712787

[b27] Narayanan K. *et al.* Dual functional roles of dentin matrix protein 1: Implications in biomineralization and gene transcription by activation of intracellular Ca2+ store. J Biol. Chem. 278 **(19)**, 17500–17508 (2003). 12615915 10.1074/jbc.M212700200

[b28] Kristensen L. P. *et al.* Temporal profiling and pulsed SILAC labeling identify novel secreted proteins during *ex vivo* osteoblast differentiation of human stromal stem cells. Mol Cell Proteomics. 10, 989–1007 (2012). 10.1074/mcp.M111.012138 PMC3494153 22801418

[b29] Rani S. *et al.* Isolation of exosomes for subsequent mRNA, MicroRNA, and protein profiling. Methods Mol Biol. 784, 181–195 (2011). 21898221 10.1007/978-1-61779-289-2_13

[b30] Shapiro I. M., Landis W. J. & Risbud M. V. Matrix vesicles: Are they anchored exosomes? Bone. 79, 29–36 (2015). 25980744 10.1016/j.bone.2015.05.013 PMC4501874

[b31] Mahamid J., Addadi L. & Weiner S. Crystallization Pathways in Bone. Cells Tissues Organs. 194, 92–97 (2011). 21576906 10.1159/000324229

[b32] He G., Dahl T., Veis A. & George A. Nucleation of apatite crystals *in vitro* by self-assembled dentin matrix protein 1. Nat Mater. 2, 552–558 (2003). 12872163 10.1038/nmat945

[b33] Gregory C. A., Grady Gunn W., Peister P. & Prokop D. J. An Alizarin red-based assay of mineralization by adherent cells in culture: comparison with cetylpyridinium chloride extraction. Anal Biochem. 329 **(1)**, 77–84 (2004). 15136169 10.1016/j.ab.2004.02.002

[b34] Ravindran S. *et al.* Stress chaperone GRP-78 functions in mineralized matrix formation. J. Biol. Chem. 286, 8729–8739 (2012). 10.1074/jbc.M110.179341 PMC3059005 21239500

[b35] Bedran-Russo A. K., Ravindran S. & George A. Imaging analysis of early DMP1 mediated dentine remineralization. Arch Oral Biol. 58, 254–260 (2012). 23107046 10.1016/j.archoralbio.2012.09.007 PMC3736807

[b36] He G. & George A. Dentin matrix protein 1 immobilized on type I collagen fibrils facilitates apatite distribution *in vitro*. J. Biol. Chem. 279 **(12)**, 11649–11656. (2004). 14699165 10.1074/jbc.M309296200

[b37] Ravindran S., Song Y. & George A. Development of three-dimensional biomimetic scaffold to study epithelial-mesenchymal interactions. Tissue Eng Part A. 16 **(1)**, 327–342 (2010). 19712044 10.1089/ten.tea.2009.0110 PMC2806069

[b38] Ravindran S., Zhang Y., Huang C. C. & George A. Odontogenic induction of dental stem cells by extracellular matrix-inspired three-dimensional scaffold. Tissue Eng Part A. 20 **(1-2)**, 92–102 (2014). 23859633 10.1089/ten.tea.2013.0192 PMC3875192

