# Supplementary material for: TGF beta receptor II interacting protein-1, an intracellular protein has an extracellular role as a modulator of matrix mineralization
Source: Sci Rep. 2016 Nov 24;6:37885. doi: 10.1038/srep37885 (PMC5121659; doi:10.1038/srep37885)
Supplement: Supplementary Information [file srep37885-s1.pdf]

TGF beta receptor II interacting protein-1, an intracellular protein has an extracellular role as a modulator of matrix mineralization

Amsaveni Ramachandran, Sriram Ravindran, Chun-Chieh Huang and Anne George*

Brodie Tooth Development Genetics & Regenerative Medicine Research Laboratory, Department of Oral Biology, University of Illinois at Chicago, Chicago, Il 60612, USA

* To whom correspondence should be addressed.

Tel.: 312-413-0738; Fax: 312-996-6044; E-mail: anneg@uic.edu

Supplementary Figure S1:

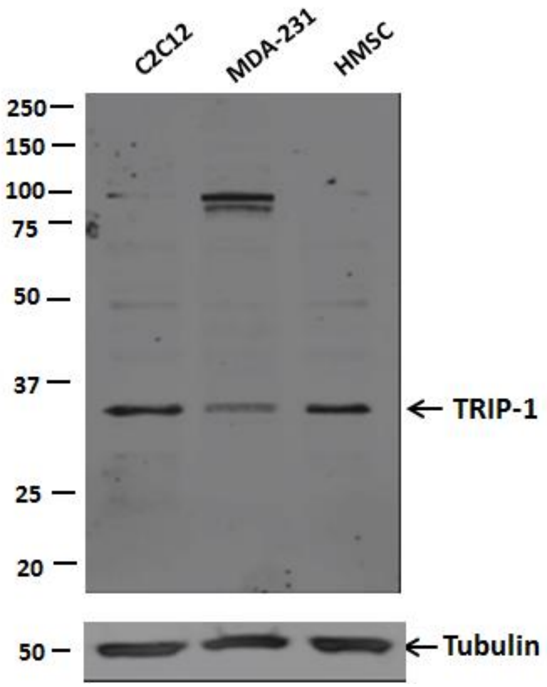

Figure S1: Western blot analysis of TRIP-1 expression in total cell lysates from C2C12, MDA-231 and HMSCs. Expression of TRIP-1 was similar in C2C12 and HMSCs, while MDA-231 cells showed lesser expression.

Supplementary Figure S2

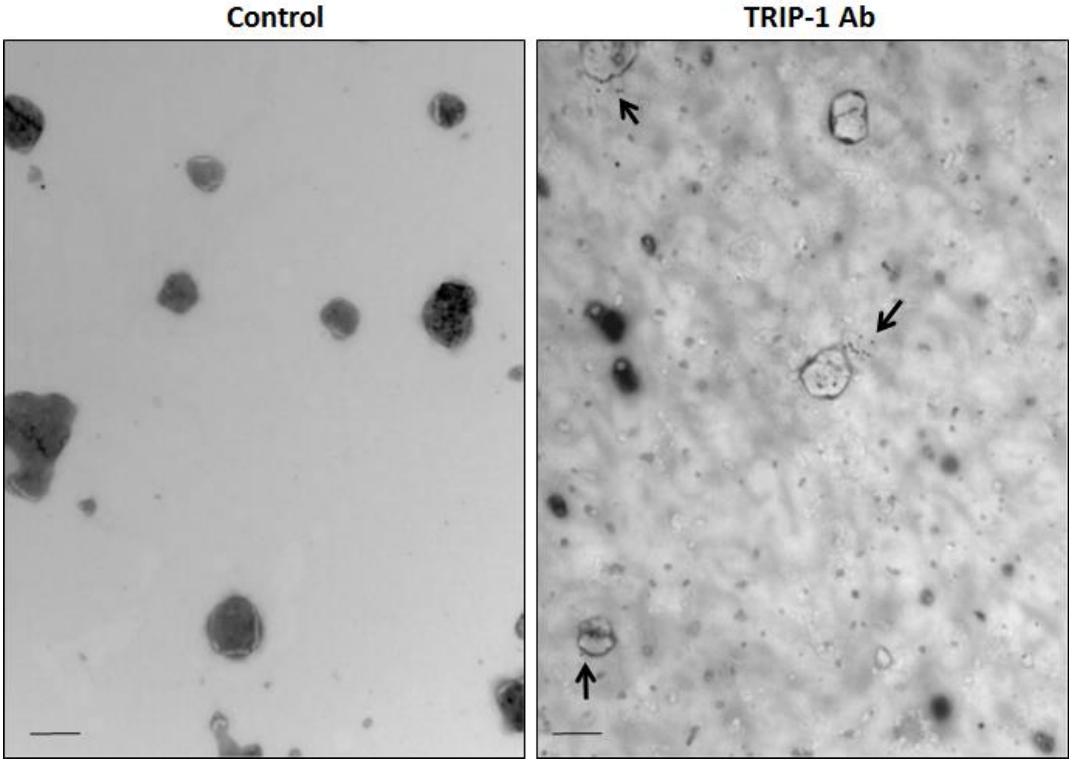

Figure S2: Representative TEM images of control and TRIP-1 labeled gold nanoparticles (10 nm, black arrows) on the cross-sectional surface of dentinal tubules. Scale bar= 0.5 μm.

Supplementary Figure S3

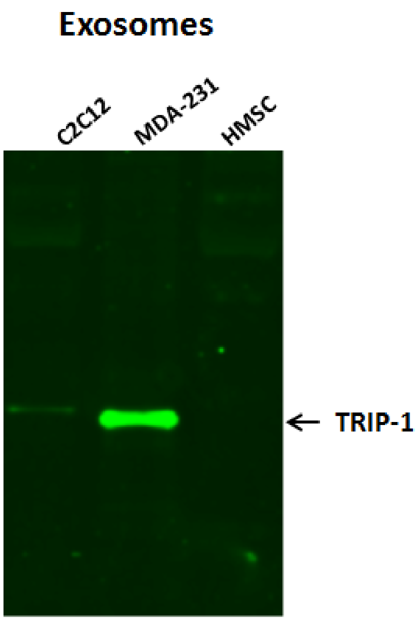

Figure S3: Western blot analysis of TRIP-1 expression in exosome fractions isolated from C2C12, MDA-231 and HMSCs. Expression of TRIP-1 was less in C2C12 exosomes while MDA-231 exosomes showed an increase in expression. TRIP-1 expression was not observed in HMSCs.

Supplementary Figure S4

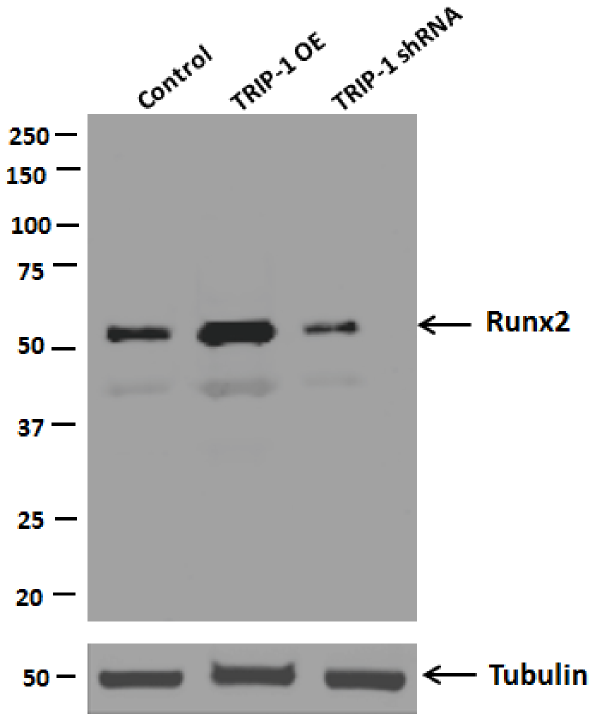

Figure S4: Western blot analysis of Runx2 expression from MC3T3 Control, MC3T3 TRIP-1 overexpressing and MC3T3 TRIP-1 shRNA cells. Expression of Runx2 was increased in TRIP-1 OE cells when compared to control cells while TRIP-1 shRNA cells showed reduced expression.

Supplementary Figure S5

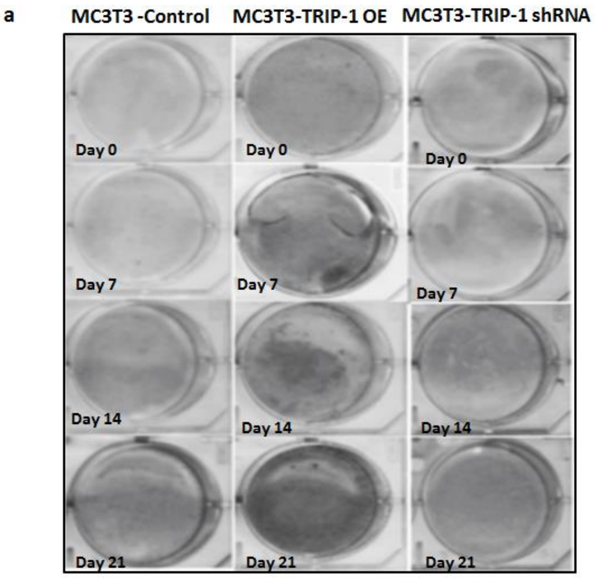

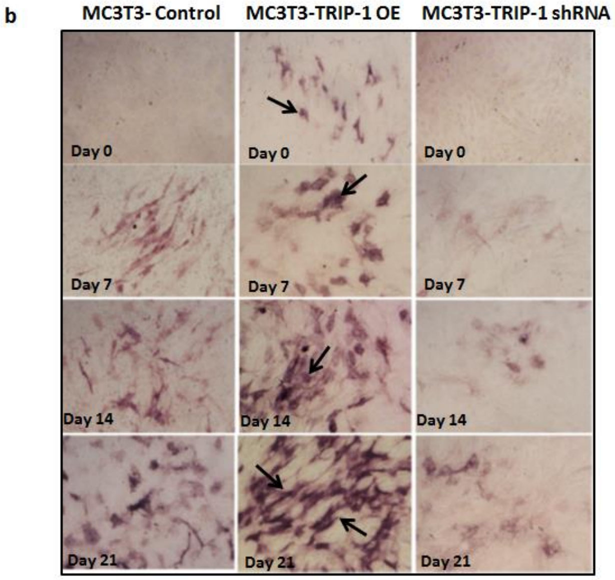

Figure S5: **S5a:** Representative scanned images of the von Kossa staining on cells grown up to 21days. **S5b:** ALP assay showing increased alkaline phosphatase expression up to 21 days in TRIP-1 overexpressing cells when compared to control cells. TRIP-1 silenced cells showed a reduction in alkaline phosphatase activity. Scale bar = 100 μm.

Supplementary Figure S6

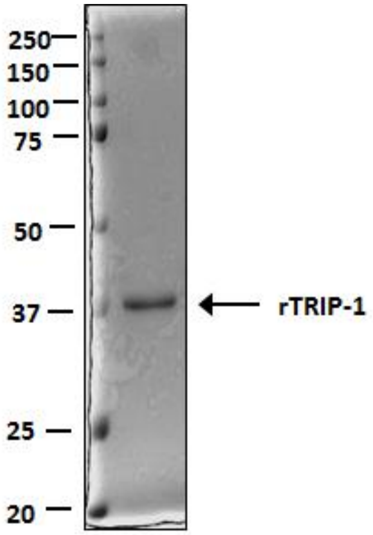

Figure S6: SDS-PAGE gel showing the purified recombinant TRIP-1 at 37 kDa.

Supplementary Figure S7

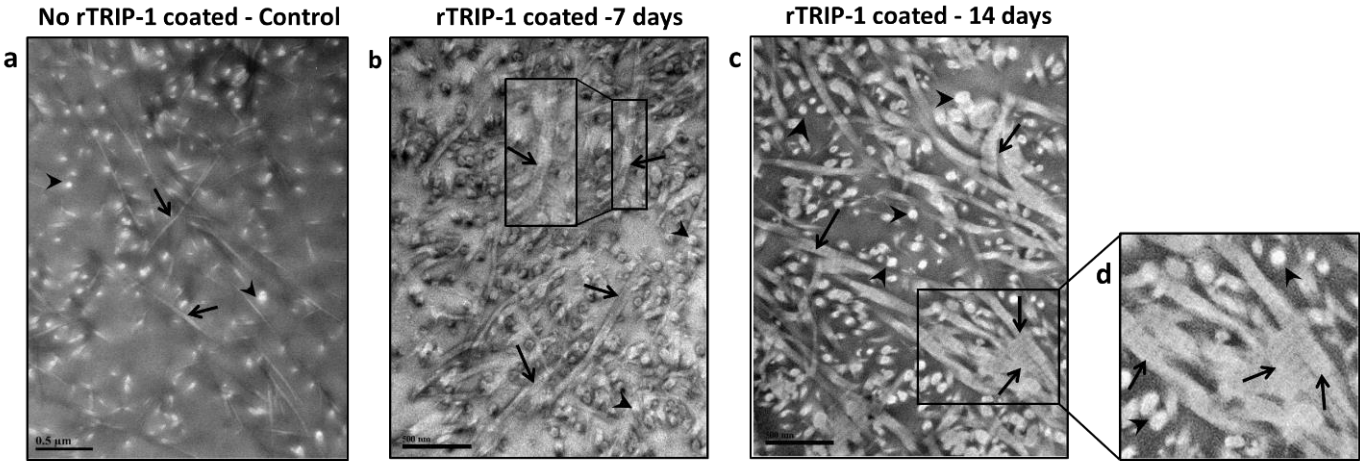

Figure S7: Representative Transmission Electron Micrographs of the dentin sections coated with rTRIP-1 following demineralization and deproteinization. **S7b** and **S7c** depict the representative unstained TEM images of 100 µg rTRIP-1 coated demineralized dentin wafer subjected to 7 and 14 days *in vitro* nucleation respectively, under physiological calcium and phosphate concentrations. Mineralized collagen fibrils were clearly observed on rTRIP-1 coated dentin matrix (black arrows) when compared to control dentin wafers (**S7a**). Inset in **S7b** shows the digital magnification of boxed area containing mineralized collagen fibril. **S7d** shows the digital magnification of the boxed area in figure **S7c.** Black arrow heads point to mineral deposits.

Supplementary Figure S8

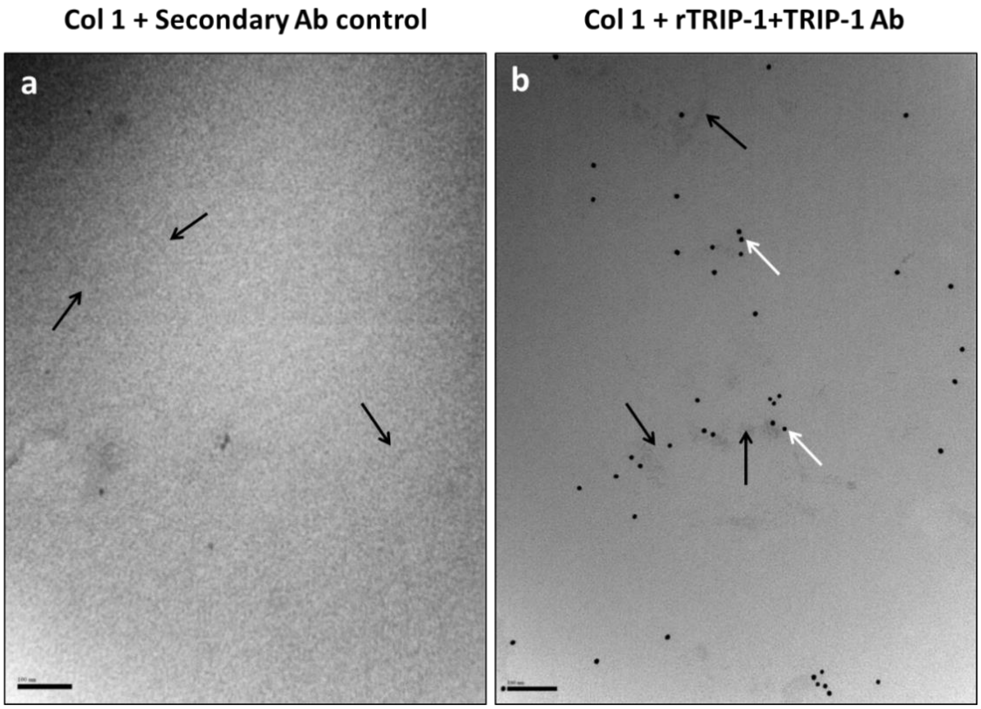

Figure S8: TEM micrograph of monomeric collagen incubated with recombinant TRIP-1 protein and labeled with TRIP-1 antibody. Control has no rTRIP-1 protein **(S8a)**. Black Arrows point to collagen fibrils and white arrows **(S8b)** indicate the gold particles labeling rTRIP-1 protein.
